# Challenging the pipeline

**DOI:** 10.1016/j.stemcr.2021.08.004

**Published:** 2021-09-14

**Authors:** Peter Loskill, Rhiannon N. Hardwick, Adrian Roth

**Affiliations:** 1 Department of Biomedical Engineering, Faculty of Medicine, Eberhard Karls University Tübingen, Tübingen, Germany; 2 NMI Natural and Medical Sciences Institute at the University of Tübingen, Reutlingen, Germany; 3 3R-Center for *In vitro* Models and Alternatives to Animal Testing, Eberhard Karls University Tübingen, Tübingen, Germany; 4 Translational Safety Sciences, Theravance Biopharma US, Inc., South San Francisco, CA, USA; 5 Personalized Healthcare Safety Interface, Product Development Safety, Roche Innovation Centre Basel, Basel, Switzerland

## Abstract

This commentary presents a thought experiment seeking to answer the key question: “If you were to put aside all the traditional drug discovery processes and start a new drug discovery program that places the highest priority on human and disease-relevant models throughout the entire process, how could it be done?”

## Teaser

In this commentary, Loskill and colleagues present a thought experiment seeking to answer the key question: “If you were to put aside all the traditional drug discovery processes and start a new drug discovery program that places the highest priority on human and disease-relevant models throughout the entire process, how could it be done?”

## Main text

The increasing availability of patient-derived human cells, the emergence of induced pluripotent stem cells (iPSCs), spheroids, organoids, and organ-on-chip models over the last decade have yielded a repertoire of an entirely new class of human-relevant *in vitro* models. Accompanied by the high aspiration to induce a paradigm change in drug development, these novel technologies came with sometimes bold claims and weighty promises—yet, thus far only a few concrete success stories have surfaced that would support this vision. The literature is, however, rich in assessments of the shortcomings of the traditional drug development paradigm (Khanna, 2012; Kola and Landis, 2004; Paul et al., 2010; Scannell et al., 2012) as well as in proof-of-concept studies demonstrating the application of these novel models for different areas of drug discovery, e.g., mechanistic issue resolution in toxicology studies, aspects of disease modeling, and pharmacokinetic analyses in models representative of single organ systems (Ewart et al., 2018; Kopec et al., 2021; Marx et al., 2020). While these have been encouraging examples that underpin the potential of such innovative human *in vitro* systems, the widespread routine application across the drug discovery pipeline in drug companies or even broader adoption of models across the pharmaceutical industry has yet to be realized. Several reasons limiting implementation of these novel technologies have been cited (e.g., lack of robustness of technology for use in industrial settings, unclear added value over existing approaches, paucity of industry-derived published datasets due to intellectual property limitations, concerns about regulatory acceptance, minimal knowledge on translatability to a clinical outcome, etc.); however, there are two major aspects hampering the adoption of these novel models:

(1) *Lack of adequate qualification and clear benchmarking:* Understanding model performance with respect to a specific context of use is critical to determining its potential for application. This requires thorough assessment of data reproducibility, human relevance of chosen endpoints for a given question, as well as solid qualification and characterization of cells used, to name just a few. However, most of the models that are published are highly exploratory in nature often using limited sets of molecules, a minimal number of human donors, and application in a single laboratory. In addition, many of these novel technologies require dedicated skills and are very labor intensive; both of these aspects may trigger significant upfront investment decisions that—based on the points raised above—are difficult to take. Collectively, these considerations lead end-users to question whether implementation of the model is truly value-adding with a sufficient return on investment.

(2) *Habit and risk aversion:* The pharmaceutical industry business, being under enormous pressure to constantly innovate under competitive conditions, budget, and time restrictions, may hesitate to change established drug development pathways if there is no sufficiently demonstrated benefit. Therefore, pharma companies can appear slow in changing and conservative regarding adoption of new technologies. This is particularly true for toxicology, where the need for conservative safety assessment is in the best interest of the patient and failure comes at an unacceptably high price for society. Technology providers and academic researchers who typically work on very specific applications often also under-estimate the holistic nature of drug safety assessment that integrates numerous different aspects from various data sources ranging from toxicology and pharmacology to pathology to drug metabolism and pharmacokinetics and to formulation sciences, for a full assessment of hazard potential and associated risk for patients. Naturally, this can result in a conservative approach and additional data from, e.g., one new type of *in vitro* model in such a complex setting may have only little impact. The reluctance to change traditional practices thus arises from uncertainty in the reliability of a model to predict true human response.

Although there are obviously a number of technical challenges to be solved, it is not our intent to cover aspect (1) in detail, since there has been a large number of reviews on the current state and challenges of organoid and organ-on-chip models over the last years (e.g., Low et al., 2021; Marx et al., 2020; Probst et al., 2018; Wikswo et al., 2013; Zhang et al., 2018). We rather would like to focus on aspect (2) and provide our perspective of how to address habit and risk aversion.

## A strategic reboot of the drug discovery process

It is commonly anticipated that if the bold claims and promises around these novel technologies are eventually substantiated, their implementation in the drug discovery process will require a transitory, “phased in” approach. This transition can either occur by sequential one-to-one replacement of individual models and assays already in use, or by boldly introducing an entirely new framework for the entire pipeline right from the beginning onward with a strategy to validate the framework “baked in.” The former approach is certainly the most straightforward and conservative, and has already been initiated in a number of instances. Yet, it is arguably a slow, “low gain” approach with only incremental impact on the overall drug discovery process and does not take full advantage of the benefits by this new class of human *in vitro* models. The alternative approach, however, does require a strategic reboot, a rethinking and smart transition concept to achieve a shift in confidence and adoption.

**Figure 1:**
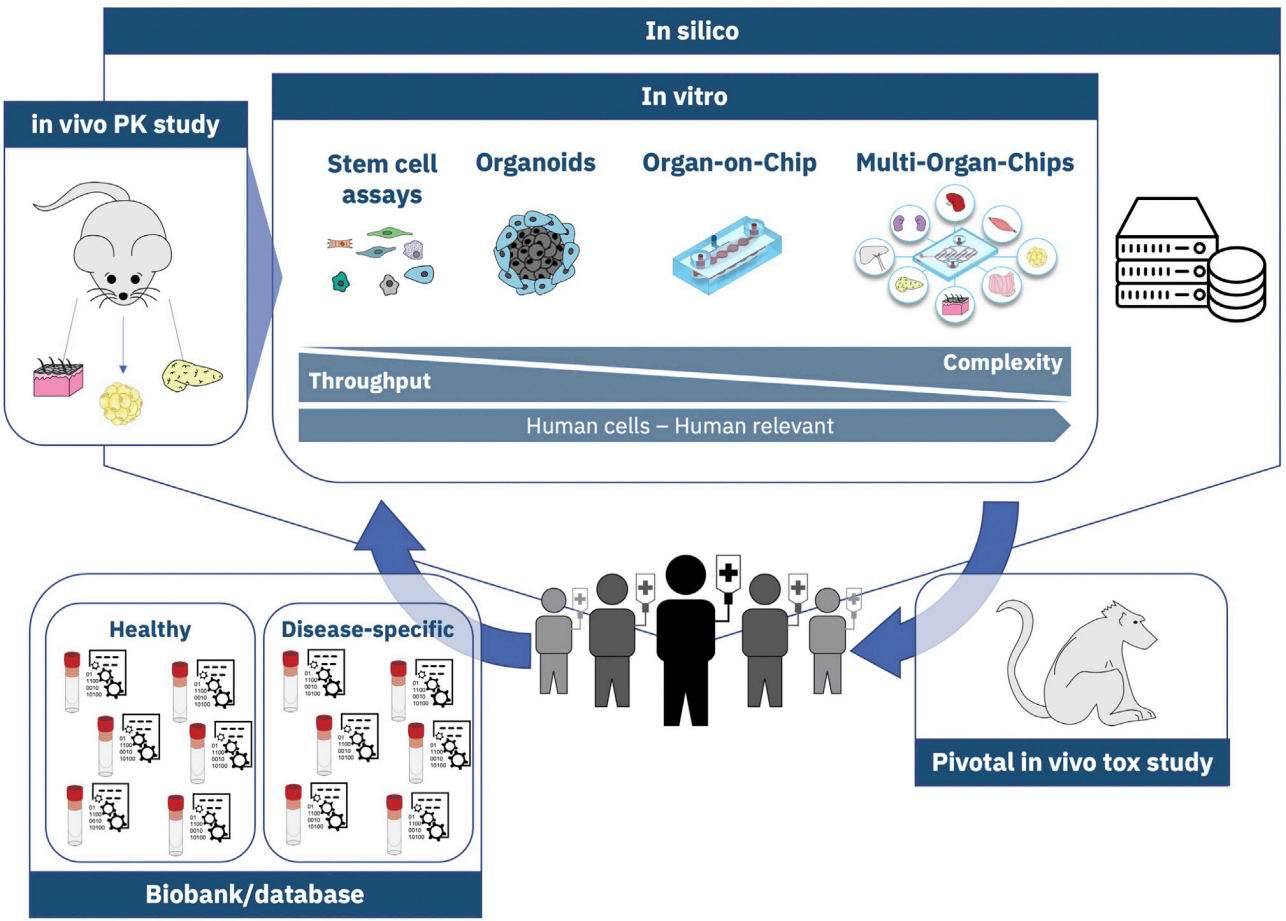
Schematic concept of a possible alternative approach for a new drug discovery program

With this in mind, we began a thought experiment seeking to answer the key question: “If you were to put aside all the traditional drug discovery processes and start a new drug discovery program that places the highest priority on human and disease-relevant models throughout the entire process, how could it be done?” (cf. Figure 1).

HiPSC technology and big data approaches could be combined to generate a stem cell biobank and a database with real world data of “representative humans” (physical and digital avatars). Importantly, this biobank/database should encompass a general healthy control group broadly applicable for all programs but also specific patient groups representing relevant disease types with a continuously expanding diversity of diseases. Obviously, as for large-scale biobank and database initiatives in general, challenges such as licensing, availability, data ownership, privacy, ethics, commercial use of data, and materials would need to be addressed. Here, experience and implemented solutions from previous and on-going efforts will be beneficial (Díaz-Zuccarini et al., 2016; Mallon et al., 2013). This biobank/database could then serve as a basis for the entire pipeline supplying the complete toolbox of models ranging from high-throughput capable, simple stem cell assays via organoids and organ-on-chip all the way to complex, low-throughput multi-organ-chip models. Furthermore, such a shift in the drug discovery approach would require merging iPSC, organoid, and organ-on-chip technologies with some of the more traditional, simpler *in vitro* assays and *in vivo* studies in a strategic, informative manner. Several relatively simple assays are anticipated to remain as stalwarts of the drug discovery pipeline due to their proven utility as underlined by years of use and thus comparative data between the pre-clinical model and true clinical outcomes (e.g., metabolism in liver microsomes, mutagenicity in bacterial strains, phototoxicity in fibroblasts, hERG assay for risk of arrhythmias, etc.). However, to usher in greater reliance on new technologies, *in vivo* pharmacokinetic studies could be utilized early in the drug discovery process to inform distribution of the investigational therapeutic as a means of identifying key organ systems in which to assess potential for toxicity. Toxicity studies could then be conducted via *in vitro* models representative of the target distribution organs, as well as those relevant to the intended route of administration, alongside pharmacodynamic analyses in the therapeutic target organ(s). *In silico* modeling approaches could help to fill data gaps due to the limited number of tissues assessed by loading up front for predictive purposes and on the back end for full integration of data across the various models/assays used to better inform human dose prediction (Myatt et al., 2018; Schmidt et al., 2014; Shi and Zha, 2019). Collectively, this approach would aim at incorporating higher human relevance as well as disease relevance earlier in the drug discovery process. A therapeutic window may be estimated and further assessed from the beginning on by parallel assessment of efficacy and safety in healthy and diseased model systems. This would be particularly impactful for diseases in which *in vivo* pre-clinical models are not available or are insufficient. In addition, one could then determine not only the impact of the investigational therapeutic within the disease environment, but also gain a deeper understanding of how human disease impacts pharmacokinetics and toxic responses. Finally, the current pivotal *in vivo* pre-clinical toxicology study in one of the species required for registration could be conducted in a confirmatory manner (on- and off-target organs, exposures, no adverse effect doses) just prior to initiation of first-in-human studies, addressing also potential signals not captured by the *in vitro* tests before.

## Implementation of a novel pipeline concept

Obviously, it is much easier to propose novel concepts and conduct thought experiments than getting such a bold change to the current approach in drug discovery implemented in the fast-paced and highly regulated environment of pharmaceutical R&D. To build trust and confidence in any new approach, the generation of datasets demonstrating real back- and forward-translation and identification of strengths and limitations will be crucial. Hence, it is of utmost importance to conduct so-called test runs where, e.g., both the traditional and novel approaches run in parallel. Another critical consideration when taking a non-traditional approach to drug discovery is to identify the pathway with the highest likelihood of success. With that in mind, two not entirely independent options are proposed:

*Option 1—focus on discrete areas of high unmet medical need:* rare and/or orphan diseases offer an extraordinary opportunity to approach drug discovery in a non-traditional manner. Rare diseases are those that occur in a small percentage of the population, namely fewer than 200,000 as defined by the US Orphan Drug Act. They are more generally thought of as neglected diseases that have not been substantially addressed with therapeutic intervention due to lack of knowledge and understanding of the disease leading to an imbalance in cost for drug development versus potential return on investment due to low patient population. To address this challenge, regulatory agencies have offered incentive programs to pharmaceutical developers to encourage research and therapeutic development. In addition, patient advocacy groups organized around rare diseases have increased patient and sample accessibility to further facilitate research. Due to their rare nature and often human-specific clinical presentation, rare diseases represent what is typically considered a challenge to drug developers, but in light of the lack of representative pre-clinical models could be viewed perhaps also as an opportunity for developers of novel technologies. This is where a non-traditional drug discovery approach could have considerable impact as options to use *in vivo* pre-clinical models typically are limited and the need for more human- and disease-relevant models is even higher compared to more common diseases (e.g., diabetes, chronic obstructive pulmonary disease, rheumatoid arthritis, etc.). Furthermore, direct collaboration with patient advocacy groups to access patient data and tissue samples could serve as the basis for an iPSC-based biobank as described above. One could then envision a more personalized approach to drug discovery wherein patient involvement continues through to clinical trials. As opposed to simply providing samples to enable the search for an effective therapeutic for their disease, which is often an abstract concept for the patient, this non-traditional, personalized approach, such that patients have more direct insight and also perceived ownership to the drug development process, is likely to increase public/private engagement.

*Option 2—adopting a “shadow pre-clinical program”*: besides rare diseases, it is important to gather data on the value and feasibility of a new drug discovery approach also for other indications as well as other types of modalities. Here, a second potential implementation option could be explored that mitigates associated risks: a shadow pre-clinical program. What does that mean? It could be a voluntary or obligatory program that requires (and potentially funds) pharmaceutical companies that successfully submit an Investigational New Drug (IND) application to (partially) "re-run" their pre-clinical data package with the alternative approach in parallel to the conventional clinical studies (i.e., phase 1). This option obviously involves a significant financial investment and will need to carefully balance the interests of the various stakeholders involved. However, the direct comparison of the novel approach with conventional programs and most importantly results from the clinic, potentially even on patient-specific levels, promises huge benefits for public health as well as pharmaceutical companies. Collaborations with technology developers could both help model refinement, as well as serve as an incentive for the pharmaceutical industry to invest in such a path if program specific and financial risks are balanced by regulatory and public support.

## Outlook

Fundamental discoveries and developments in engineering and biology, as well as the merging of the two disciplines, cell biology and micro-engineering, have brought us into the fortunate situation in which we have access to a new class of human *in vitro* models. While it is crucial to balance expectations, since these young technologies still face big challenges and need for refinement, these models undoubtedly have significant potential. Hence, it is important to bring all relevant stakeholders together and ensure that everyone, on the one hand, gains a thorough understanding of processes, needs, as well as limiting factors of the individual stakeholders and, on the other hand, engages with an open mind while shelving habit and risk aversion. Here, we present a thought experiment that by no means claims to be definite and complete; yet, it is meant to plant a seed to think about ideas and concepts on how to change the way we discover and develop drugs beyond the often proposed one-by-one replacement of models/assays. We are convinced that bold visions and strategies are required and that it is also crucial to form public-private partnerships that discuss, define, and, most importantly, execute concrete implementation strategies.
